# Comparative Proteomic and Morphological Change Analyses of *Staphylococcus aureus* During Resuscitation From Prolonged Freezing

**DOI:** 10.3389/fmicb.2018.00866

**Published:** 2018-05-03

**Authors:** Biao Suo, Hua Yang, Yuexia Wang, Haipeng Lv, Zhen Li, Chao Xu, Zhilu Ai

**Affiliations:** ^1^College of Food Science and Technology, Henan Agricultural University, Zhengzhou, China; ^2^Key Laboratory of Staple Grain Processing, Ministry of Agriculture, Zhengzhou, China; ^3^Henan Engineering Laboratory of Quick-Frozen Flour-Rice Food and Prepared Food, Henan Engineering Research Center for Cold-Chain Food, Henan Agricultural University, Zhengzhou, China

**Keywords:** frozen food, sublethal injury, resuscitation, proteomics, HPLC-MS, differentially expressed protein

## Abstract

When frozen, *Staphylococcus aureus* survives in a sublethally injured state. However, *S. aureus* can recover at a suitable temperature, which poses a threat to food safety. To elucidate the resuscitation mechanism of freezing survived *S. aureus*, we used cells stored at -18°C for 90 days as controls. After resuscitating the survived cells at 37°C, the viable cell numbers were determined on tryptic soy agar with 0.6% yeast extract (TSAYE), and the non-injured-cell numbers were determined on TSAYE supplemented with 10% NaCl. The results showed that the total viable cell number did not increase within the first 3 h of resuscitation, but the osmotic regulation ability of freezing survived cells gradually recovered to the level of healthy cells, which was evidenced by the lack of difference between the two samples seen by differential cell enumeration. Scanning electron microscopy (SEM) showed that, compared to late exponential stage cells, some frozen survived cells underwent splitting and cell lysis due to deep distortion and membrane rupture. Transmission electron microscopy (TEM) showed that, in most of the frozen survived cells, the nucleoids (low electronic density area) were loose, and the cytoplasmic matrices (high electronic density area) were sparse. Additionally, a gap was seen to form between the cytoplasmic membranes and the cell walls in the frozen survived cells. The morphological changes were restored when the survived cells were resuscitated at 37°C. We also analyzed the differential proteome after resuscitation using non-labeled high-performance liquid chromatography–mass spectrometry (HPLC-MS). The results showed that, compared with freezing survived *S. aureus* cells, the cells resuscitated for 1 h had 45 upregulated and 73 downregulated proteins. The differentially expressed proteins were functionally categorized by gene ontology enrichment, KEGG pathway, and STRING analyses. Cell membrane synthesis-related proteins, oxidative stress resistance-related proteins, metabolism-related proteins, and virulence factors exhibited distinct expression patterns during resuscitation. These findings have implications in the understanding of the resuscitation mechanism of freezing survived *S. aureus*, which may facilitate the development of novel technologies for improved detection and control of foodborne pathogens in frozen food.

## Introduction

*Staphylococcus aureus* is a leading cause of gastroenteritis resulting from the consumption of contaminated food (Hennekinne et al., 2012). Freezing is widely used to ensure the safety and quality of food. However, previous research has shown that low temperatures are sublethal to *S. aureus*; at low temperatures, the *S. aureus* cell itself is damaged, but not dead (Suo et al., 2014). The sublethally injured cells are hard to detect because of their sensitivities to selective components, such as high concentrations of sodium chloride, that are widely used in selective enrichment media (Wu, 2008). Moreover, when the contaminated frozen food is placed at suitable temperatures, the sublethally injured cells are resuscitated and are able to proliferate again; hence, *S. aureus* poses a severe threat to food safety (Wesche et al., 2009).

When foodborne pathogens undergo a sublethal injury under environmental stress, such as during freezing, several structural and functional components are affected; the cell membrane appears to be the component most commonly affected (Wu, 2008; Wesche et al., 2009). A previous study reported that cold stress induced a high percentage of small colony variants (SCVs) when *S. aureus* was subjected to a temperature of 4°C for a prolonged period. SCVs exhibited ultra-structural changes including increased cell wall thickness (Onyango et al., 2012). In contrast to the 4°C condition, freezing causes more damage to the cell membrane because of the formation of ice crystals (Archer, 2004). Damage to the cell membrane leads to the leakage of cellular content, the disruption of membrane permeability, and the weakening of the osmoregulation ability of the cell (Wesche et al., 2009; Suo et al., 2014). This damage also reduces the fluidity of the cell membrane and reduces the enzyme activity, according to a study on *Listeria monocytogenes* (Schmid et al., 2009). Therefore, repair of the cell membrane must occur relatively rapidly upon alleviation of adverse conditions to allow the cells to recover from stress-induced lesions.

Freezing temperatures harm gene transcription and translation, leading to the inhibition of cell replication. After the stress of freezing temperature has been alleviated, appropriate signal transduction pathways in the surviving cells must be activated immediately to repair the damage. To investigate the possible mechanism of resuscitation, several researchers have used proteomic analysis to examine the influence of environmental stress on genome-wide translational expression in foodborne pathogens. Vidovic et al. (2011, 2012) analyzed wild-type *Escherichia coli* O157 and the *rpoS* mutant strain at low temperatures through comparative proteomics. Their results showed that the differentially expressed protein (DEP) RpoS could regulate related protein expression under cold stimulation. Cacace et al. (2010) revealed that *L. monocytogenes* was able to survive at low temperatures by synthetizing large amounts of glycolytic enzymes and activating energy-producing metabolic pathways. Using proteomic assays, Rivas et al. (2013) revealed a significant change in the expression of some structural and metabolic proteins when *E. coli* was resuscitated after pulsed electric field processing. A proteomic and metabolomic study of *S. aureus* in response to prolonged cold stress showed that the survival of cold-induced SCVs was closely associated with the regulatory mechanisms of metabolic homeostasis and protein composition, such as the upregulation of glycolysis (Alreshidi et al., 2015). Their findings indicated that the resuscitation of sublethally injured cells was tightly related to the proteins involved in cell structure and metabolism. However, so far, there is no report describing the resuscitation mechanism in *S. aureus* cells that have survived freezing.

This study implemented the non-labeling high-performance liquid chromatography–mass spectrometry (HPLC-MS) technique to analyze the differential proteome of *S. aureus* cells resuscitated after freezing. The DEPs were classified and the function of each protein category was predicted. Our research can provide a basis for further analysis of the resuscitation mechanism of freezing survived *S. aureus*.

## Materials and Methods

### *S. aureus* Culture Conditions and Freezing Treatment

*Staphylococcus aureus* ATCC6538 of clinical origin, which was obtained from American Type Culture Collection (ATCC), was used in this study and stored at -80°C with glycerol. We selected ATCC6538 for this study because we have previously quantified the survival kinetics and gene transcriptional responses of this strain to cold stress, and we know that this strain can survive for a long time at freezing temperatures (Suo et al., 2014, 2015). For the culture, we inoculated the *S. aureus* into 100 mL tryptic soy broth with 0.6% yeast extract (TSBYE). After incubation at 37°C for 6 h with shaking at 150 rpm, 1 mL of the late exponential phase culture (approximately 10^9^ CFU/mL) was transferred into a 1.5-mL Eppendorf tube. The cells were washed twice with 0.1% peptone water and resuspended in 0.1 mL TSBYE. In total, 200 tubes were prepared and then quick-frozen (-40°C, 30 min, air velocity 8 m/s; HJLSY-II Quick-freezing machine, Henry Refrigeration Equipment Co., Ltd., Zhengzhou, China) and stored in a -18°C refrigerator. After 90 days of frozen storage, the cells were retrieved directly for use. According to our previous report (Suo et al., 2014), *S. aureus* can survive under this condition for longer than 90 days, and the viable cell number will not decrease any further.

### Resuscitation of Freezing Survived Cells

After 90 days of frozen storage, three vials of *S. aureus* samples were retrieved from the refrigerator. After adding 0.9 mL of TSBYE broth pre-warmed at 37°C, the 1-mL culture was immediately transferred to a thermostatic bath (Thermal Fisher Scientific, United States) pre-set at 37°C under agitation (150 rpm). At predetermined intervals, the cultures were retrieved for differential plate counts.

### Viable and Non-injured Cell Enumeration on Differential Plates

The viable and non-injured cells were enumerated using differential plate counting methods as described in our previous report (Suo et al., 2014). Non-selective tryptic soy agar with 0.6% yeast extract (TSAYE) and selective TSAYE (supplemented with 10% NaCl) agars were used to determine the total viable cell count and the non-injured cell count, respectively. The 6 × 6 method was used for the viable cell count (Chen et al., 2003). The TSAYE and TSAYE supplemented with 10% NaCl plates were incubated aerobically at 37°C for 24 and 48 h, respectively, prior to counting the viable cells.

### Scanning Electron Microscopy

The cell morphology of *S. aureus* after 90 days of storage at -18°C and 2 h of resuscitation at 37°C was compared with late exponential phase cells as described in our previously reported method (Suo et al., 2017). The cells were washed twice using phosphate-buffered saline (PBS) and fixed overnight with 4% glutaraldehyde. After removing the residual glutaraldehyde by washing in PBS, the cells were fixed a second time in 2% glutaraldehyde. Subsequently, the samples were washed again using the same PBS, followed by dehydration with a series of ethanol solutions (20, 50, 80, and 100%; v/v), with two changes of the ethanol solution at each concentration. The dehydrated cell sample was dispersed on a 1 cm × 1 cm piece of aluminum-foil paper and freeze-dried with a vacuum freeze dryer (ES-2030, Hitachi, Ltd., Japan). Then, the samples were sputter coated with a thin layer of gold using an E-1010 ion-sputtering apparatus (Hitachi, Ltd., HQ, Japan) prior to scanning electron microscopy (SEM) observation. Digital images were acquired using an S-3400N-II scanning electron microscope (Hitachi, Ltd., HQ, Japan) at an instrumental magnification of 10,000×.

### Transmission Electron Microscopy

For transmission electron microscopy (TEM) analysis, the cells from each treatment were harvested and washed three times with PBS and fixed for 4 h at 4°C with 4% (v/v) glutaraldehyde. The cells were washed four times in the same PBS and then fixed for 1.5 h by using 1% osmium tetroxide. The samples were dehydrated sequentially in a series of acetone solutions (30, 50, 70, 90, and 100%; v/v) and then embedded in an embedding medium (Epon 812) for 4 h. The ultrathin sections were stained with uranyl acetate and lead citrate for 10 min and were observed through a JEM-1400 transmission electron microscope (JEOL Japan Electronics Co., Ltd., Japan).

### Extraction and Purification of the Total Protein

To investigate proteins related to resuscitation, we used freezing survived cells of *S. aureus* as a control to compare their proteomic pattern to that of resuscitated cells; the technique used for this comparison was non-labeled HPLC-MS quantitative proteomic analysis. The total protein was extracted from the survived *S. aureus* control cells after 90 days of storage at -18°C and from the resuscitated cells after 1 h of incubation at 37°C. Cells were harvested by centrifugation of 5 mL of bacterial culture. After triple washing with PBS, the bacterial pellet was resuspended in lysis buffer (100 mM Tris HCl, 2% SDS, 100 mM DTT, and 100 μL of a cocktail of proteinase inhibitors; pH 7.6), and incubated for 5 min at 95°C. The solution was then sonicated for 30 min on ice (with 200 W power). One milliliter of the suspension was centrifuged (16,100 rpm, 10 min) to obtain the supernatant, which was used for the measurement of protein concentration with a BCA Assay Kit as a reference (Pierce, IL, United States). The samples were then added by 200 μL trypsin, as well as 1 μL DTT at a final concentration of 0.5 M. After incubation at 57°C for 20 min, the samples were added by iodoacetamide to achieve a final concentration of 5.5 mM. The tubes were placed in the dark for 15 min at room temperature, prior to the addition of 1 μL of 1% (m/v) surfactant (ProteasMAX, Promega, Madison, WI, United States) and 1.8 μL of l μg/μL trypsin (TrypsinGold, Promega). The mixture was digested at 37°C for 12 h to produce the final trypsinized peptide sample. The samples were then acidified with 4 μL of 10% (v/v) formic acid (FA) and stored at -20°C until HPLC-MS analysis. The protein was extracted from three biological replicates of each sample. With the purpose of further minimizing the deviation between different samples under the same treatment, the sample of each biological replicate was retrieved from five independent culture vials.

### Peptide Separation and Identification by Non-labeled HPLC-MS

The fraction was separated by nano-HPLC (Eksigent Technologies) on a secondary RP analytical column (Eksigent, C18, 3 μm, 150 mm × 75 μm). Peptides were subsequently eluted using the following gradient conditions with phase B (98% acetonitrile with 0.1% FA) from 5 to 45% (5–100 min). The total flow rate was maintained at 300 nL/min. The electrospray voltage of 2.5 kV versus the inlet of the mass spectrometer was used.

Triple TOF 4600 mass spectrometer was operated in an information-dependent data acquisition mode to switch automatically between MS and MS/MS acquisition. MS spectra were acquired across a mass range of 350–1250 *m/z*, using an accumulation time of 250 ms per spectrum. The tandem mass spectral scanned from 100 to 1250 *m/z* in high sensitivity mode with rolling collision energy. The 25 most intense precursors per cycle were selected for fragmentation with a dynamic exclusion time of 25 s.

### Database Search, Protein Identification, and Expression Quantification

The non-labeled quantitative analysis software program Progenesis QI. Proteomics 1.0 (Nonlinear Dynamics) was utilized to analyze the samples based on MS peak abundance. The total quality was filtered with a 1% false discovery rate (FDR). After the quantitative analysis of protein expression, spectrograms were used for protein identification using Peaks Studio 7.0 software by querying the *S. aureus* COL strain database, which contains 2680 protein sequences. Based on quantification results for three biological replicates of each sample, Student’s *t*-test was used to evaluate the significance for the DEPs and to evaluate the fold change between the two samples. Those with |log2 fold change| > 2 and *P* < 0.05 were deemed upregulated or downregulated proteins, respectively.

The DEPs were functionally categorized according to the gene ontology (GO) annotation by BLAST2GO software^1^. The Kyoto Encyclopedia of Genes and Genomes (KEGG)^2^ was used to predict the pathways of the DEPs. Protein–protein interactions (PPIs) were predicted by the Search Tool of the Retrieval of Interaction Genes/Proteins (STRING) database^3^ (Szklarczyk et al., 2015), and the interaction network was illustrated by Cytoscape software (Bachman et al., 2012). The PPIs with interaction scores higher than 0.4 (medium confidence) were subjected to further interaction network analysis.

## Results and Discussion

### Resuscitation Pattern of Freezing Survived Cells of *S. aureus*

**FIGURE 1:**
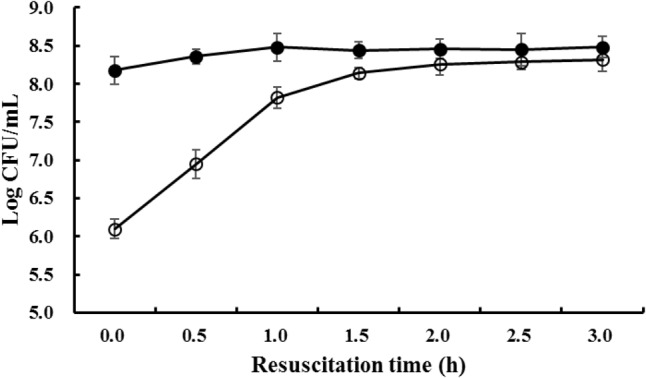
The viable cell counts of *S. aureus* when resuscitated from prolonged freezing. The black bar represents the total viable cell number enumerated on TSAYE agar and the white bar represents the non-injured cell number enumerated on TSAYE agar supplemented with 10% NaCl.

The viable cell numbers of freezing survived *S. aureus* during resuscitation were counted on both TSAYE medium and TSAYE medium supplemented with 10% NaCl. As shown in **Figure 1**, after storage at -18°C for 90 days, the viable *S. aureus* cell numbers determined from the two kinds of culture media have a difference of 2log CFU/mL. This result proves that a majority of the viable cells are sublethally injured after prolonged freezing (Suo et al., 2014). The difference in cell growth between the two kinds of agars can be attributed to the formation of ice crystals within frozen sublethally injured cells; these ice crystals can harm the cell membrane (Archer, 2004) and lead to the reduced capability of osmotic regulation (Mackey, 2000).

When the freezing survived cells were resuscitated at 37°C, the difference in viable cell numbers between the two kinds of media gradually decreased, and after 3 h, the difference in viable cell numbers between the two media was almost indistinguishable (*P* > 0.05). This result indicated that, during resuscitation, the cell membranes of freezing survived *S. aureus* cells were gradually repaired, and as a result, the osmotic regulation capability recovered to the normal level.

### Morphological Changes of *S. aureus* During Resuscitation

**FIGURE 2:**
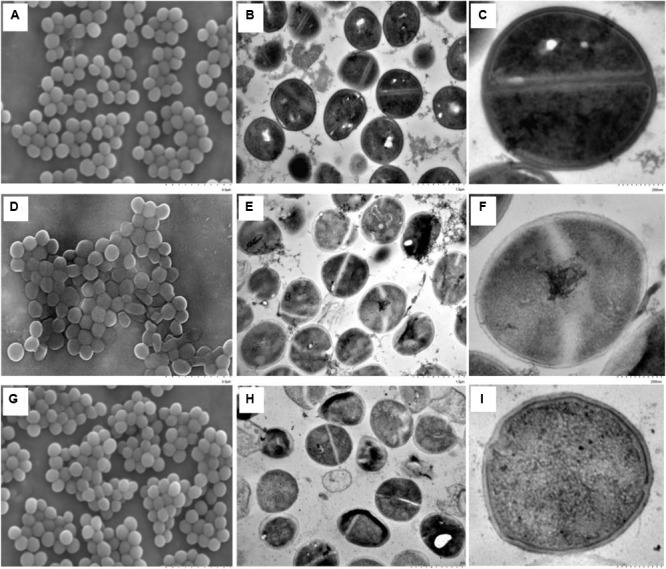
Morphological changes of *S. aureus* during resuscitation from prolonged freezing. The scanning electron microscopic images **A, D**, and **G** were observed at an instrumental magnification of 10,000×. The transmission electron microscopic images **B, E**, and **H** were observed at an instrumental magnification of 30,000×, while **C, F**, and **I** were observed at an instrumental magnification of 100,000×. **(A–C)** The exponential-phase cells. **(D–F)** Cells that have been stored at –18°C for 90 days. **(G–I)** Resuscitated cells cultured in TSBYE at 37°C for 2 h.

Changes in the cell morphology of *S. aureus* during resuscitation from freezing condition were observed by both SEM and TEM analyses. The late exponential phase cells, grown for up to 6 h, were used as the control, because these cells were considered mature; cells at the stationary phase of growth, after longer incubation, were considered physiologically old (Truong et al., 2017). As shown in the SEM observation, smooth cell envelopes of normal cells were observed when *S. aureus* cells were grown to the late exponential phase (**Figure 2A**). However, some cells that underwent prolonged freezing exhibited splitting and a high degree of cell lysis, which were caused by deep distortion and membrane rupture (**Figure 2D**). The changes in cell morphology were assumed to be the result of impaired cell division (Onyango et al., 2012). The morphological changes of *S. aureus* caused by prolonged freezing condition could be restored after 2 h of resuscitation at 37°C (**Figure 2G**), at which point almost no difference in cell morphology was observed compared to late exponential phase cells.

Ultrastructural changes of *S. aureus* during resuscitation from freezing condition were observed by TEM. As shown in **Figure 2**, compared to late exponential phase cells, the nucleoids (low electronic density area) in most of the freezing survived cells were loose, and the cytoplasmic matrix (high electronic density area) was sparse. There was almost no combination of dark granules and nucleic acid material in the nucleoids of the freezing survived cells. Additionally, a gap was seen to form between the cytoplasmic membrane and the cell wall in the freezing survived cells (**Figure 2F**). A similar result of membrane–wall separation has also been observed by TEM in *S. aureus* under cinnamaldehyde treatment (Shen et al., 2015) and under mild thermal treatment (Li et al., 2017). In contrast, under a low-temperature treatment at 4°C, *S. aureus* cells had significantly thicker and more diffuse cell walls than their corresponding control samples (Onyango et al., 2012).

When the freezing survived cells were resuscitated for 2 h, the cytoplasmic matrices of the resuscitated cells were more densely stained than those of the freezing survived cells (**Figures 2H,I**). The results were coincident with the TEM observation of resuscitated *E. coli* after high-pressure CO_2_ treatment, which was attributed to the increase in the number of ribosomes (Zhao et al., 2013). Several dark granules were seen to be in combination with nucleic acid material. This combination could promote the accumulation of cell components within a short time, which would accelerate cell growth and multiplication (Nilsson et al., 2002; Takamatsu et al., 2016). Moreover, fewer cells exhibit a gap between the cytoplasmic membrane and the cell wall. After resuscitation, the cell surface appeared compact and sturdy compared with the smooth and transparent appearence of the freezing survived cells.

Taking into account the differences between total viable and non-injury cell numbers, the morphological differences, and the differences in the interior characteristics of the resuscitated (2 h) and freezing survived cells, the following observation can be made: the total viable cell number did not change although the number of 10%-NaCl-tolerant cells increased to the level of total viable cells. Therefore, the SEM and TEM observations in this study suggested that the resuscitation was a unique process and was not caused by the proliferation of non-injured cells (Zhao et al., 2013).

### Resuscitating *S. aureus* Has 45 Upregulated and 73 Downregulated Proteins Compared to Freezing Survived Cells

To elucidate the possible molecular mechanism behind the resuscitation, we used non-labeled HPLC-MS quantitative proteomic analysis to compare the proteomic pattern of freezing survived *S. aureus*, as the control, to that of cells obtained after 1 h of resuscitation at 37°C. The resuscitating (1 h) cells have also been used as materials to investigate the resuscitation mechanism in pulsed electric field treated *E. coli* by proteomic analysis (Rivas et al., 2013). Based on the MS analysis of the quantification results for three biological replicates of each sample, the expression levels of the DEPs were verified for statistical significance using Student’s *t*-test. The present study identified 975 proteins from 10,836 peptides at an FDR of 1% (Supplementary Tables S1, S2), where 118 showed a greater than twofold difference in expression with a *p*-value less than 0.05 (Supplementary Table S3). Among the DEPs, 45 proteins (38.1%) have a max fold change value higher than two, indicating the significant increase in expressions of these proteins during resuscitation initiation, whereas 73 proteins (61.9%) have a max fold change value less than 0.5, showing the significant decrease of protein expression during resuscitation.

### Functional Categorization of DEPs

**FIGURE 3:**
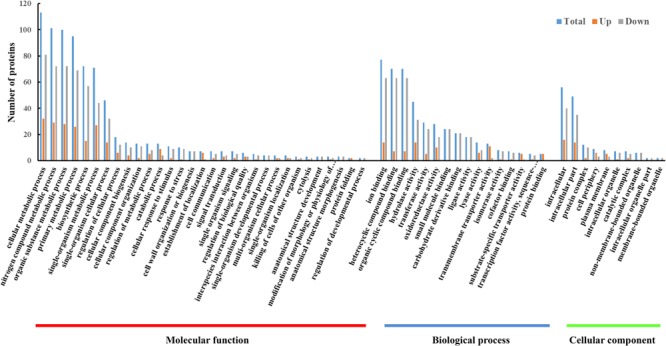
GO enrichment analysis of DEPs between resuscitating and freezing survived control cells of *S. aureus*. The *x*-axis represents different GO terms. The *y*-axis represents the number of proteins classified as being involved in biological processes, molecular functions, and cellular components, shown until the third level of complexity.

To understand the gene functions associated with the DEPs, the GO enrichment analysis was performed using BLAST2GO software. We classified the upregulated and downregulated proteins based on the relative categories annotated by the GO method (**Figure 3**). The GO functional categorization generated 1365 annotations from the 118 DEPs; 359 of these annotations were generated from upregulated proteins and 1006 annotations were generated from downregulated proteins. Among these, at the first level of classification, 767, 440, and 158 DEPs were classified as being involved in molecular functions, biological processes, and cellular components, respectively. Among those classified as being involved in molecular function, 113, 101, 100, and 95 DEPs were classified into the categories of cellular metabolic process (GO:0044237), nitrogen compound metabolic process (GO:0006807), organic substance metabolic process (GO:0071704), and primary metabolic process (GO:0044238), respectively. The results indicated that the DEPs were predominantly metabolism-associated. Among the DEPs classified as being involved in biological process, a majority were binding related, including 77 ion binding (GO:0043167), 70 heterocyclic compound binding (GO:1901363), and 70 organic cyclic compound binding (GO:0097159) DEPs. Among the DEPs classified as being involved in cellular component, those involved in intracellular (GO:0005622) and intracellular part (GO:0044424) constituted the largest proportion. The GO analysis showed that the DEPs were associated with various functions involving different molecular functions, biological processes, and cellular components.

### KEGG Pathway Analysis of DEPs

**FIGURE 4:**
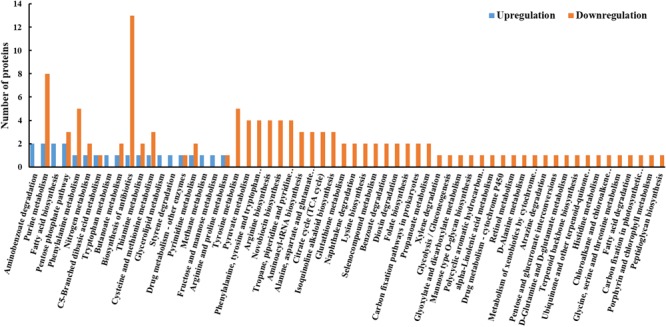
KEGG pathway analysis of DEPs between resuscitating and freezing survived control cells of *S. aureus*. The *x*-axis represents different KEGG pathways. The *y*-axis represents the number of proteins.

The functions of the DEPs were further analyzed by the KEGG pathway annotation method and 58 pathways were obtained, including 19 upregulated pathways and 53 downregulated pathways (**Figure 4**). The five most enriched pathways were pathways involved in biosynthesis of antibiotics, purine metabolism, phenylalanine metabolism, and tyrosine metabolism, and the pentose phosphate pathway.

### Protein–Protein Interactions of DEPs

**FIGURE 5:**
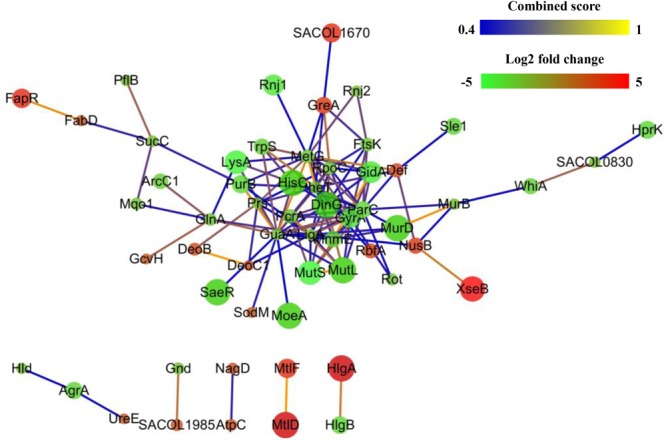
Protein–protein interaction networks of differentially expressed proteins (DEPs) of resuscitating *S. aureus* cells compared to freezing survived cells. Proteins are indicated with nodes, and interactions between proteins are represented by edges. The edge colors indicate the combined score. The node colors represent upregulated protein (red) or downregulated protein (green), and the size of node also indicates the change in the level of protein expression.

The PPIs of the DEPs were analyzed using the STRING online search tool. As shown in **Figure 5**, PPIs with interaction scores higher than 0.4 were used to further build a network using Cytoscape software. The result indicated that the PPI network of resuscitating cells vs. freezing survived cells contained 71 nodes and 127 edges. The number of edges is higher than the expected number of edges (108), indicating that the proteins have more interactions among themselves than what would be expected for a random set of proteins of similar size drawn from the genome. Such an enrichment indicates that, as a group, the DEPs are at least partially biologically connected.

### Regulation of Cell Membrane Synthesis-Related Proteins During Resuscitation

Under freezing conditions, the cell membrane is damaged by ice crystals; this is the main reason for sublethal injury and even death of cells (Archer, 2004). Membrane damage also leads to the reduced cellular capability for osmotic regulation (Mackey, 2000) and to sensitivity toward higher concentration of NaCl, as evidenced by the results in **Figure 1**. Therefore, cell membrane repair is supposed to be one of the most important procedures that occur during resuscitation.

It has been reported that the purine biosynthetic pathway plays an important role in cell wall synthesis, and the entire purine biosynthetic pathway was shown to undergo powerful and reversible repression under conditions where the cell wall synthesis was perturbed and slowed down (Sobral et al., 2007). The proteomic results in this study indicated that eight proteins involved in purine metabolism were inhibited significantly. The results suggested that the purine metabolism pathway was suppressed in *S. aureus* during resuscitation from freezing condition; this suppression was beneficial for maintaining the purine content of the cell in order to meet the increased need for ATP due to the excess cell wall material produced (Mongodin et al., 2003).

As shown by the proteomic analysis, some cell membrane synthesis-related proteins are upregulated, including lactonase Drp35 (Drp35), malonyl CoA-acyl carrier protein transacylase (FabD), and fatty acid biosynthesis transcriptional regulator (FapR). Drp35 participates in the synthesis of the cell membrane of *S. aureus*, allowing the cell to survive in harsh conditions (Tanaka et al., 2007). FabD is an NADH-dependent component that increases the synthesis of cell membrane fatty acids (Hong et al., 2010). FabD also showed a greater abundance in tea tree oil-selected *S. aureus* SCVs (Torres et al., 2017). FapR is a transcriptional factor participating in bacteria membrane synthesis. FapR removes obstacles in fatty acid synthetic pathways through negative regulation of the *fap* operon (Schujman et al., 2003). It is known that fatty acids are the main components of cell membranes, which play important roles in stress resistance (Sado-Kamdem et al., 2009). Changes in fatty acid content affect cell membrane surface enzymes and receptor function through their influence on membrane fluidity (Kulp and Kuehn, 2010). The upregulated proteins promote the synthesis and transport of fatty acids. Thus, these proteins help to repair damage caused by ice crystals and assist in the maintenance of osmolarity during cell resuscitation.

### Regulation of Superoxide Dismutase (SodM) During Resuscitation

There are some reactive oxygen species (ROS) generated during aerobic respiration that damage the DNA, protein, and lipids within cells. Proteins associated with oxidative stress response have been shown to be present at higher abundance in both nisin-treated (Miyamoto et al., 2015) and cold-adapted *L. monocytogenes* cells (Cacace et al., 2010). Superoxide dismutase (SodM) bound to Mn^2+^and Fe^2+^ in the cytosol protects bacterial macromolecules from ROS damage (Valderas and Hart, 2001). The upregulation of SodM had been shown to play a great role in modulating oxidative stress resistance in *S. aureus* (Ballal and Manna, 2009). Increasing level of this protein maintains the stabilities of nucleic acids, proteins, and lipids, thereby facilitating the resuscitation of freezing survived *S. aureus*.

### Regulation of Metabolism-Related Proteins During Resuscitation

Adaption to a resuscitating environment after a prolonged freezing condition requires a shift in the expression of proteins involved in specific metabolic pathways. In the present study, the freezing survived *S. aureus* was resuscitated in TSBYE with agitation. In an oxygen-rich environment, glucose and other sugars are processed by the citrate cycle rather than by fermentation and the pentose phosphate pathway. Therefore, it was reasonable that glucose-6-phosphate 1-dehydrogenase (encoded by the *zwf* gene) and 6-phosphogluconate (encoded by the *gnd* gene) both exhibited decreased expression (Supplementary Table S3), because they both are the restrict enzymes in the pentose phosphate pathway (Panosian et al., 2011). Three citrate cycle-related proteins, namely, malate:quinone oxidoreductase (encoded by the *mqo1* gene), pyruvate carboxylase (encoded by the *pyc* gene), and sucinyl-coA ligase subunit beta (encoded by the *sucC* gene), also exhibited downregulated expression. Proteomics analysis determined that the citrate cycle in *L. monocytogenes* was also inhibited in response to cold stress (Cacace et al., 2010; Pittman et al., 2014).

Interestingly, mannitol-specific phosphotransferase enzyme IIA component (encoded by the *mtlF* gene) and mannitol-1-phosphate 5-dehydrogenase (encoded by the *mtlD* gene) both showed an increase in expression during resuscitation. The MtlF protein is present in the cytosol and is one of the key components of the phosphotransferase system (PTS) in Gram-positive bacteria. The PTS system is responsible for the transport of carbohydrates (Fischer and Hengstenberg, 1992). The increased expression of PTS-associated proteins indicated an enhanced requirement for sugar uptake, which was in agreement with the results of a proteomic study of the response of *L. monocytogenes* to low temperature stress (Cacace et al., 2010; Pittman et al., 2014). The induction of PTS proteins implies a need for the production of complex macromolecules and energy during resuscitation after prolonged freezing. MtlD mainly participates in fructose and mannose metabolism by inducing the formation of mannitol-1-phosphate from fructose-6-phosphate. Mannitol can then be formed by the catalytic action of phosphatase, and be utilized as a carbon source for metabolism (Reshamwala et al., 2014). Given that TSBYE is a complex medium for resuscitation, carbohydrates other than glucose are available to be transported into the cell and metabolized. The increased expression of MtlF and MtlD implies a specific metabolism that utilizes fructose and mannose as carbon sources or as compatible solutes during resuscitation, especially when the citrate cycle and pentose phosphate pathways are both restricted.

### Regulation of Virulence-Related Proteins During Resuscitation

The pathogenicity of *S. aureus* is one of the most important issues faced by food producers and consumers. The expression of the virulence-related protein delta-hemolysin (Hld) was inhibited during the resuscitation of freezing survived *S. aureus*. By activating the mast cells of the host, Hld causes itchiness, which leads to scratching, during *S. aureus* infection (Hodille et al., 2016). Predictably, the accessory gene regulator A (AgrA), which controls the expressions of Hld (Hodille et al., 2016), is also downregulated during resuscitation. This result suggests that the expression of Hld and AgrA during resuscitation after a prolonged freezing is closely associated. Gamma-hemolysin components A (HlgA) and B (HlgB) are both leukotoxins of *S. aureus* (Otto, 2014), but our results show that they were regulated differentially during resuscitation, as evidenced by the observation that the expression of HlgA is significantly upregulated but that of HlgB is downregulated. The serine-aspartate repeat protein D (SdrD) was induced during resuscitation. The SdrD protein is important for the adhesion of *S. aureus* to host cells and increases the virulence and survival in blood of *S. aureus* (Askarian et al., 2017). A previous report showed that the SdrD protein was downregulated in response to prolonged cold stress (Alreshidi et al., 2015). The upregulation of the SdrD protein in the present study implies that the resuscitating cells have prepared for invasion upon alleviation of the freezing conditions.

Interesting, the S-ribosylhomocysteine lyase (LuxS) and AgrA proteins showed upregulation and downregulation, respectively. These are both quorum-sensing-related proteins and have been shown to play important roles in many biological processes, including biofilm formation and host infection (Le and Otto, 2015). The different regulatory patterns of LuxS and AgrA indicated that they participate independently in functional regulation, which is in agreement with a previous gene mutant analysis (Doherty et al., 2006). Moreover, the gene mutant analysis indicated that in addition to the regulation of virulence factors, LuxS also plays a role in metabolism (Doherty et al., 2006). The results from the present study indicated that LuxS might play a role different from that of AgrA in assisting *S. aureus* to physiologically adapt to the altered requirements during resuscitation from freezing conditions.

Although only one *S. aureus* strain was used in the present study, the proteomics data offer a comprehensive view for understanding the resuscitation mechanism of freezing survived cells. However, it should be noted that the results obtained using this representative strain cannot be relevant for all the common variants of *S. aureus*. Because strain-specific protein repertoires are involved in many biological processes (Liang et al., 2016), it seems unlikely that there is only one resuscitation mechanism for freezing survived cells. Moreover, the present proteomics analysis was performed using cells directly exposed to freezing but not in a frozen food sample. In real conditions of frozen food, the food matrices may affect positively or negatively the recovery of *S. aureus*, especially when the contamination level is normally lower in real food comparing with the inoculation in a rich nutrient niche of broth. To guarantee the bacterial safety of frozen food, further research is still necessary for a complete understanding of the resuscitation mechanism of freezing survived cells in the context of their interactions with food ingredients.

## Conclusion

In the present study, when *S. aureus* cells survive freezing condition, the cell envelope is seen to be damaged. This damage manifests as the loss of osmolarity regulation due to high NaCl concentration, and the damage can also be directly observed using SEM and TEM. When the freezing survived cells were transferred to a 37°C environment, the sublethally injured cells gradually recovered to normal. We used a quantitative non-labeled HPLC-MS technique to compare the abundances of proteins during resuscitation. Our results revealed differences in protein expression between resuscitating and freezing survived cells, providing new insight into resuscitation mechanisms. These results lay the foundation for understanding the survival and adaptation biology of *S. aureus* in frozen food and should facilitate the development of novel recovery and enrichment assays. Eventually, this study could lead to rapid detection techniques and to the development of novel frozen-food-specific control methods to supplement the currently available prevention measures to control *S. aureus*.

## Data Availability

The datatsets supporting the conclusions of this article are included within the article and its additional files. The mass spectrometry proteomics data of this articile are available in iProX (Integrated Proteome resources) and the data set identifier is IPX0001087000.

## Author Contributions

BS and ZA conceived and designed the experiments. BS, HY, and HL performed the experiments. ZL contributed reagents/materials/analysis tools. BS, YW, and HL analyzed the data and wrote the paper. CX and ZA critically revised the manuscript. All authors read and approved the final version of the manuscript.

## Conflict of Interest Statement

The authors declare that the research was conducted in the absence of any commercial or financial relationships that could be construed as a potential conflict of interest.

## Footnotes

1 https://www.blast2go.com/

2 http://www.kegg.jp/kegg/

3 https://string-db.org/
