# Supplementary material for: Comparative Proteomic and Morphological Change Analyses of Staphylococcus aureus During Resuscitation From Prolonged Freezing
Source: Front Microbiol. 2018 May 3;9:866. doi: 10.3389/fmicb.2018.00866 (PMC5943506; doi:10.3389/fmicb.2018.00866)
Supplement: Supplementary file 3 [file Table_3.DOCX]

**Supplementary Table S3 |** List of the differentially expressed proteins (DEPs) between resuscitating *S. aureus* and freezing survived control (|log2 fold Change| > 2.0, *P* < 0.05)

|  | **Protein description** | **NCBI accession no.** | **Gene name** | **Change (fold)** | ***p*-Value** |
| --- | --- | --- | --- | --- | --- |
| **Upregulated protein** | | | | |  |
| 1 | 6-phosphogluconolactonase | Q5HEJ8 | SACOL1985 | 2.311 | 0.038 |
| 2 | Aldo keto reductase family oxidoreductase | Q5HEZ1 | SACOL1835 | 3.234 | 0.008 |
| 3 | Alpha-acetolactate decarboxylase | Q5HDZ8 | *aldC* | 2.066 | 0.005 |
| 4 | Anti-sigma-B factor antagonist | P60071 | *rsbV* | 2.495 | 0.001 |
| 5 | Arginine/ornithine antiporter | Q5HCR4 | *arcD* | 142.30 | 0.015 |
| 6 | ATP synthase epsilon chain | Q5HE98 | *atpC* | 2.269 | 0.020 |
| 7 | ComE operon protein 2 | Q5HFH2 | *comEB* | 3.449 | 0.027 |
| 8 | Deoxyribose-phosphate aldolase 1 | Q5HJN0 | *deoC1* | 2.011 | 0.002 |
| 9 | Dihydroxyacetone kinase subunit DhaK | Q5HI18 | *dhaK* | 2.592 | 0.012 |
| 10 | Exodeoxyribonuclease 7 small subunit | Q5HFP9 | *xseB* | 4.809 | 0.002 |
| 11 | Fatty acid biosynthesis transcriptional regulator fapR | Q5HGK5 | *fapR* | 3.699 | 0.022 |
| 12 | Gamma-hemolysin component A (leukotoxin) | Q5HDD6 | *hlgA* | 6.628 | 0.002 |
| 13 | Gluconate transporter, permease protein | Q5HD49 | *gntP* | 5.227 | 0.049 |
| 14 | Glycine cleavage system protein H | Q5HHK8 | *gcvH* | 2.036 | 0.003 |
| 15 | HAD family hydrolase | Q5HHF6 | *nagD* | 2.154 | 0.016 |
| 16 | HIT family protein | Q5HET7 | *hit* | 3.960 | 0.049 |
| 17 | Lactonase Drp35 | Q5HCK9 | *drp35* | 6.405 | 0.001 |
| 18 | LuxR family DNA-binding response regulator | Q5HG99 | *bvgA* | 2.741 | 0.032 |
| 19 | M20/M25/M40 family peptidase | Q5HFR1 | *pepT* | 4.368 | 0.030 |
| 20 | Magnesium and cobalt transport protein corA | Q5HDH8 | SACOL2375 | Infinity* | 0.001 |
| 21 | Malonyl CoA-acyl carrier protein transacylase | Q5HGK3 | *fabD* | 2.099 | 0.003 |
| 22 | Mannitol-1-phosphate 5-dehydrogenase | Q9RL68 | *mtlD* | 25.856 | 0.031 |
| 23 | Mannitol-specific phosphotransferase enzyme IIA component | Q5HE46 | *mtlF* | 3.655 | 0.016 |
| 24 | Molybdate ABC periplasmic molybdate-binding protein ModA | Q5HDS8 | *modA* | 2.336 | 0.001 |
| 25 | N-acetylmuramoyl-L-alanine amidase | Q5HCQ3 | SACOL2666 | 2.147 | 0.001 |
| 26 | Nitrite reductase [NAD(P)H] small subunit | Q5HDF7 | *nirD* | 2.513 | 0.044 |
| 27 | Oxidoreductase ion channel | Q5HI67 | *iolS* | 3.157 | 0.001 |
| 28 | Peptide deformylase | Q5HGZ3 | *def* | 2.715 | 0.017 |
| 29 | Phosphopentomutase | Q5HJM9 | *deoB* | 2.458 | 0.016 |
| 30 | Ribosome-binding factor A | Q5HGG1 | *rbfA* | 2.835 | 0.024 |
| 31 | Serine-aspartate repeat-containing protein D | Q5HIB3 | *sdrD* | 4.680 | 0.002 |
| 32 | S-ribosylhomocysteine lyase | Q5HE66 | *luxS* | 3.600 | 0.006 |
| 33 | Superoxide dismutase [Mn/Fe]2 | Q5HJN5 | *sodM* | 2.039 | 0.031 |
| 34 | Thioredoxin | Q5HEJ1 | SACOL1992 | 3.953 | 0.002 |
| 35 | Transcription antitermination factor NusB | Q5HFP7 | *nusB* | 3.079 | 0.017 |
| 36 | Transcription elongation factor GreA | Q5HFF2 | *greA* | 3.215 | 0.001 |
| 37 | Transcriptional regulator | Q5HIR0 | SACOL0457 | 2.312 | 0.001 |
| 38 | Urease accessory protein UreE | Q5HDR7 | *ureE* | 2.030 | 0.009 |
| 39 | Uncharacterized | Q5HF22 | - | 3.462 | 0.016 |
| 40 | Uncharacterized | Q5HFZ5 | - | 2.398 | 0.017 |
| 41 | Uncharacterized | Q5HGE0 | - | 2.507 | 0.019 |
| 42 | Uncharacterized | Q5HGS2 | - | 2.134 | 0.039 |
| 43 | Uncharacterized | Q5HE32 | - | 2.931 | 0.005 |
| 44 | Uncharacterized | Q5HIA7 | - | 2.774 | 0.005 |
| 45 | Uncharacterized | Q5HFE7 | SACOL1670 | 3.637 | 0.014 |
| **Downregulated protein** | | | | |  |
| 46 | 2-C-methyl-D-erythritol 4-phosphate cytidylyltransferase | Q5HJC5 | *ispD1* | -7.246 | 0.015 |
| 47 | 6-phosphogluconate dehydrogenase | Q5HFR2 | *gnd* | -2.278 | 0.007 |
| 48 | ABC transporter ATP-binding protein | Q5HG28 | *yheS* | -2.222 | 0.004 |
| 49 | Accessory gene regulator A | Q5HEG2 | *agrA* | -3.289 | 0.023 |
| 50 | Adenylosuccinate lyase | Q5HEL4 | *purB* | -3.472 | 0.001 |
| 51 | Allophanate hydrolase subunit 2 | Q5HHV5 | SACOL0777 | -3.731 | 0.006 |
| 52 | ATP-dependent DNA helicase PcrA | Q5HEL7 | *pcrA* | -2.740 | 0.005 |
| 53 | Bifunctional acetaldehyde-CoA/alcohol dehydrogenase | Q5HJM2 | *adhE* | -2.445 | 0.003 |
| 54 | Carbamate kinase 1 | Q5HGR2 | *arcC1* | -2.967 | 0.035 |
| 55 | CMP-binding-factor 1 | Q5HET3 | *cbf1* | -3.876 | 0.023 |
| 56 | Cysteine desulfurase | Q5HF58 | *iscS* | -14.493 | 0.013 |
| 57 | D-alanine--poly(phosphoribitol) ligase subunit 1 | Q5HHF2 | *dltA* | -3.425 | 0.001 |
| 58 | Delta-hemolysin | Q5HEG6 | *hld* | -2.141 | 0.028 |
| 59 | Diaminopimelate decarboxylase | Q5HG20 | *lysA* | -4.484 | 0.019 |
| 60 | DNA gyrase subunit A | Q5HJZ0 | *gyrA* | -3.788 | 0.001 |
| 61 | DNA ligase | Q5HEL8 | *ligA* | -2.188 | 0.010 |
| 62 | DNA mismatch repair protein MutL | Q5HGD5 | *mutL* | Infinitesimal* | 0.001 |
| 63 | DNA mismatch repair protein MutS | Q5HGD6 | *mutS* | -4.587 | 0.005 |
| 64 | DNA polymerase I | Q5HF83 | *polA* | -4.975 | 0.001 |
| 65 | DNA polymerase III subunit epsilon/ATP-dependent helicase DinG | Q5HFW8 | *dinG* | -5.556 | 0.039 |
| 66 | DNA topoisomerase 4 subunit A | Q5HG64 | *parC* | -3.247 | 0.003 |
| 67 | DNA translocase FtsK | Q5HGF5 | *ftsK* | -2.632 | 0.031 |
| 68 | DNA-binding response regulator SaeR | Q5HHW4 | *saeR* | -5.882 | 0.012 |
| 69 | DNA-directed RNA polymerase subunit beta | Q5HID2 | *rpoC* | -2.545 | 0.001 |
| 70 | Epimerase | Q5HHP9 | SACOL0834 | -2.247 | 0.002 |
| 71 | Esterase | Q5HDK6 | *mlhB* | -3.717 | 0.010 |
| 72 | EVE domain | Q5HE59 | SACOL2133 | -4.367 | 0.001 |
| 73 | Exotoxin | Q5HIP9 | SACOL0468 | -3.984 | 0.040 |
| 74 | FeS assembly protein SufB | Q5HHG8 | *sufB* | -2.299 | 0.022 |
| 75 | Formate acetyltransferase | Q5HJF4 | *pflB* | -2.137 | 0.005 |
| 76 | Gamma-hemolysin component B (leukotoxin) | Q5HDD3 | *hlgB* | -3.344 | 0.001 |
| 77 | General stress Gls24 family | Q5HGK8 | SACOL1239 | -2.445 | 0.005 |
| 78 | Glucose-6-phosphate 1-dehydrogenase | Q5HFR7 | *zwf* | -2.551 | 0.001 |
| 79 | Glutamine synthetase | Q5HGC3 | *glnA* | -2.404 | 0.001 |
| 80 | Glycosyl transferase group 1 family protein | Q5HIB1 | SACOL0611 | -24.390 | 0.001 |
| 81 | Glycosyl transferase, group 1 family protein | Q5HIB0 | *tagE* | -7.463 | 0.007 |
| 82 | Glycosyl transferase, group 2 family protein | Q5HJB8 | SACOL0238 | -3.012 | 0.005 |
| 83 | GMP synthase [glutamine-hydrolyzing] | Q5HIQ6 | *guaA* | -2.257 | 0.005 |
| 84 | GntR family transcriptional regulator | Q5HIK1 | *treR* | -4.739 | 0.017 |
| 85 | Histidinol-phosphate aminotransferase | Q5HHU9 | *hisC* | -5.747 | 0.031 |
| 86 | HPr kinase/phosphorylase | Q5HHQ8 | *hprK* | -3.401 | 0.008 |
| 87 | HTH-type transcriptional regulator rot | Q5HF12 | *rot* | -2.320 | 0.023 |
| 88 | Malate:quinone oxidoreductase | Q5HDJ0 | *mqo1* | -2.381 | 0.003 |
| 89 | Methionyl-tRNA synthetase | Q5HII6 | *metG* | -2.604 | 0.001 |
| 90 | Molybdopterin molybdenumtransferase | Q5HDT4 | *moeA* | -5.236 | 0.004 |
| 91 | N-acetylmuramoyl-L-alanine amidase sle1 | Q5HIL2 | *sle1* | -3.226 | 0.001 |
| 92 | Phenylalanyl-tRNA synthetase subunit beta | Q5HGU5 | *pheT* | -2.427 | 0.001 |
| 93 | P-loop ATPase | Q5HHQ3 | SACOL0830 | -2.160 | 0.005 |
| 94 | Poly (glycerophosphate) glycerophosphotransferase family | Q5HDI8 | SACOL2364 | -8.475 | 0.008 |
| 95 | Primosomal protein DnaI | Q5HF89 | *dnaI* | -2.747 | 0.009 |
| 96 | Protoporphyrinogen oxidase | Q5HEU4 | *hemG* | -6.289 | 0.001 |
| 97 | Pyridine nucleotide-disulfide oxidoreductase | Q5HFU4 | *ypdA* | -3.367 | 0.004 |
| 98 | Pyruvate carboxylase | Q5HGX0 | *pyc* | -3.876 | 0.002 |
| 99 | Ribonuclease J 1 | Q5HGZ5 | *rnj1* | -3.984 | 0.006 |
| 100 | Ribonuclease J 2 | Q5HGF6 | *rnj2* | -2.155 | 0.004 |
| 101 | Ribose-phosphate pyrophosphokinase | Q5HIH5 | *prs* | -2.203 | 0.004 |
| 102 | Ribosomal large subunit pseudouridine synthase B | Q5HFS9 | *rluB* | -2.494 | 0.018 |
| 103 | Ribosome biogenesis GTPase A | Q5HGI9 | *rbgA* | -4.651 | 0.003 |
| 104 | RNA-binding transcriptional accessory | Q5HED8 | SACOL2053 | -5.348 | 0.002 |
| 105 | RpiR family phosphosugar-binding transcriptional regulator | Q5HDP2 | *ybbH* | -10.638 | 0.001 |
| 106 | Salicylate hydroxylase | Q5HDQ3 | *nagX* | -2.398 | 0.002 |
| 107 | Sporulation regulator C terminal domain | Q5HHQ1 | *whiA* | -3.058 | 0.001 |
| 108 | Succinyl-CoA ligase [ADP-forming] subunit beta | Q5HGI7 | *sucC* | -2.188 | 0.001 |
| 109 | Teichoic acid biosynthesis protein | Q5HJC3 | SACOL0238 | -3.356 | 0.001 |
| 110 | Teichoic acids export ATP-binding protein TagH | Q5HI31 | *tagH* | -2.513 | 0.011 |
| 111 | Transcriptional regulator DeoR family | Q5HHX1 | *fruR* | -2.193 | 0.010 |
| 112 | tRNA modification GTPase MnmE | Q5HCI3 | *mnmE* | -3.155 | 0.009 |
| 113 | tRNA uridine 5-carboxymethylaminomethyl modification enzyme | Q5HCI4 | *gidA* | -4.149 | 0.037 |
| 114 | Tryptophanyl--tRNA synthetase | Q5HH88 | *trpS* | -3.096 | 0.001 |
| 115 | Type I restriction-modification enzyme, S subunit, EcoA family | Q5HEW8 | *hsdS* | -3.731 | 0.003 |
| 116 | UDP-N-acetylenolpyruvoylglucosamine reductase | Q5HHT2 | *murB* | -2.165 | 0.001 |
| 117 | UDP-N-acetylmuramoyl-L-alanyl-D-glutamate synthetase | Q5HGP8 | *murD* | -9.901 | 0.002 |
| 118 | UPF0354 SAA6008_01714 | Q5HF31 | SACOL1793 | -6.173 | 0.008 |
